# Human β-Defensin 2 Mutations Are Associated With Asthma and Atopy in Children and Its Application Prevents Atopic Asthma in a Mouse Model

**DOI:** 10.3389/fimmu.2021.636061

**Published:** 2021-02-25

**Authors:** Natascha S. Borchers, Elisangela Santos-Valente, Antoaneta A. Toncheva, Jan Wehkamp, Andre Franke, Vincent D. Gaertner, Peter Nordkild, Jon Genuneit, Benjamin A. H. Jensen, Michael Kabesch

**Affiliations:** ^1^ Department of Pediatric Pneumology and Allergy, University Children’s Hospital Regensburg (KUNO) at Hospital St. Hedwig of the Order of St. John, Regensburg, Germany; ^2^ Department of Internal Medicine II, University Hospital Tübingen, University of Tübingen, Tübingen, Germany; ^3^ Institute of Clinical Molecular Biology (IKMB), Kiel University, Kiel, Germany; ^4^ Newborn Research Zürich, University Hospital and University of Zürich, Zürich, Switzerland; ^5^ Defensin Therapeutics ApS, Copenhagen, Denmark; ^6^ Pediatric Epidemiology, Department of Pediatrics, Medical Faculty, Leipzig University, Leipzig, Germany; ^7^ Novo Nordisk Foundation Center for Basic Metabolic Research, Faculty of Health and Medical Sciences, University of Copenhagen, Copenhagen, Denmark

**Keywords:** hBD-2, asthma, atopy, prevention, defensin

## Abstract

Asthma and allergies are complex, chronic inflammatory diseases in which genetic and environmental factors are crucial. Protection against asthma and allergy development in the context of farming environment is established by early animal contact, unpasteurized milk consumption and gut microbiota maturation. The human β-defensin 2 (hBD-2) is a host defense peptide present almost exclusively in epithelial tissues, with pronounced immunomodulatory properties, which has recently been shown to ameliorate asthma and IBD in animal models. We hypothesized that adequate hBD-2 secretion plays a role in the protection against asthma and allergy development and that genetic variations in the complex gene locus coding for hBD-2 may be a risk factor for developing these diseases, if as a consequence, hBD-2 is insufficiently produced. We used MALDI-TOF MS genotyping, sequencing and a RFLP assay to study the genetic variation including mutations, polymorphisms and copy number variations in the locus harboring both genes coding for hBD-2 (*DEFB4A* and *DEFB4B)*. We administered hBD-2 orally in a mouse model of house dust mite (HDM)-asthma before allergy challenge to explore its prophylactic potential, thereby mimicking a protective farm effect. Despite the high complexity of the region harboring *DEFB4A* and *DEFB4B* we identified numerous genetic variants to be associated with asthma and allergy in the GABRIELA Ulm population of 1,238 children living in rural areas, including rare mutations, polymorphisms and a lack of the *DEFB4A*. Furthermore, we found that prophylactic oral administration of hBD-2 significantly curbed lung resistance and pulmonary inflammation in our HDM mouse model. These data indicate that inadequate genetic capacity for hBD-2 is associated with increased asthma and allergy risk while adequate and early hBD-2 administration (in a mouse model) prevents atopic asthma. This suggests that hBD-2 could be involved in the protective farm effect and may be an excellent candidate to confer protection against asthma development.

## Introduction

Exposure to a diversity of microbes is crucial for the development and maturation of the human immune system (1–3). Children born and growing up in a traditional farm environment are protected against chronic inflammatory diseases such as asthma, allergy and inflammatory bowel diseases (IBD) (4–6), in which epithelial barrier dysfunction plays a central role (7–10). While the mechanism of protection is still elusive, associations with animal contact (11), drinking of unpasteurized milk (12) and maturation of the gut microbiota (13) were described.

Contact of microorganisms with human surfaces induce epithelial barrier defense mechanisms and the release of host defense peptides (HDPs), such as β-defensins (14–16). These are capable of eliminating a broad range of microorganisms and fine-tune the elicited immune response in multiple diseases, including IBD, cystic fibrosis, and rhinovirus infections (17–19). Genetic and/or environmental disturbances in the secretory capacity of functional HDPs may facilitate bacterial translocation (20) and simultaneously disrupt microbial homeostasis (21), collectively fueling systemic inflammation. Both aberrant inflammation and early airway colonization with microbial pathogens may predispose for childhood asthma (22). The immunomodulatory nature of many β-defensins, combined with their effective regulation of microbial colonization, positions these HDPs at center stage for a causal role in the development of inflammatory diseases and at the same time make them promising candidates for prevention and treatment of these diseases. Human β-defensin 2 (hBD-2), which is almost non-existent in the unprovoked airways and gut but profoundly abundant in inflamed epithelial tissues in response to environmental stimuli (23–26), may be of particular interest. Indeed, hBD-2 has recently been shown to ameliorate asthma and IBD in animal models (17–27).

Diverse microbial exposure in early life as well as the individual’s personal capacity to mount adequate immunological responses seem to be crucial to avoid asthma and allergy development (28). A significant part of the genetic susceptibility for asthma and allergy may be driven by variation in genes contributing to immunological processes, depending also on differences in environmental exposure (28). While recent studies on the subject have used genome-wide association study (GWAS) data, crucial regions of the genome are not covered in this approach: the genetic locus containing multiple defensin genes including hBD-2 on chromosome 8 is one of them. Due to its complex genetic architecture (29, 30) it is one of the few regions in the human genome still not fully deciphered. A further obstacle in defensin research has been the technical difficulty to produce purified defensin for experimental studies. As hBD-2 protein only became available in sufficient quantities recently, only a handful of studies have investigated the role and potential of the molecule so far (17, 27, 31, 32).

Here, we now investigated genetic variability in the extremely complex gene locus of hBD-2; performed association studies with some focus on farming environment exposure; and performed mouse experiments to explore if hBD-2 could play a role in preventing asthma development, as described in the hygiene hypothesis.

## Materials and Methods

### Human Studies

#### Study Cohort GABRIELA Ulm

To allow for the assessment of the effects of genetic variations in *DEFB4A* and *DEFB4B* on asthma and atopy in the context of farm environment exposure, we performed genetic association analyses in the GABRIEL Advanced Studies (GABRIELA) from Ulm, comprising 1,238 participants recruited in and around the city of Ulm, Germany. The primary aim of the study was to identify mechanisms of the protective farming effects on the development of asthma and atopic sensitization in primary school children from approx. 6 to 10 years of age with baseline assessment in 2006 (33, 34). Yearly follow-up assessments were conducted from 2010 to 2016 (35). A description of demographic factors is given in **Supplementary Table 1**. Asthma was defined as either reported wheeze in the past 12 months or ever inhaler use for asthma or a reported doctor’s diagnosis of asthma at least once or wheezy bronchitis at least twice throughout the lifetime. Atopy was defined as specific IgE antibodies of at least 0.35 kU/L against *Dermatophagoides pteronyssinus*, cat dander, common silver birch, or grass mix (34). The distribution of the cohort according to presence of asthma and/or atopy and farm exposure is given in **Supplementary Table 2**.

#### Single Nucleotide Polymorphism (SNP) Selection and Genotyping

To study the region on chromosome 8p23.1 containing the two almost identical genes coding for hBD-2, *DEFB4A* and *DEFB4B*, in detail and to retrieve information for already described/existing gene variants, we used publicly available online tools: Ensembl^1^, NCBI resources^2^, and the UCSC genome browser^3^. To compare the transcription factor binding sites upstream of both hBD-2 genes, we used the online tool PROMO 3.0 provided by the Algorithmics and Genetics Group (ALGGEN) in the Computer Science Department of the Universitat Politècnica de Catalunya (Barcelona, Spain). To investigate the association between SNPs in or nearby the *DEFB4A* and *DEFB4B* genes (10 kb downstream each copy and the 477kb section between them) and the development of asthma and atopy, we searched for all annotated SNPs with a minor allele frequency (MAF) ≥ 0.01 in CEU population of the 1000 Genomes project [Utah Residents (CEPH) with Northern and Western European Ancestry], which were either present in a putative regulatory (e.g. promoter region) or coding regions (or close vicinity) of the *DEFB4A* and *DEFB4B* genes. Out of 458 reported SNPs identified in the 1000 Genomes project in this region, 40 fulfilled the described criteria. For genotyping design, we used parameters (i.e. modified flank size and amplicon length), which slightly deviated from the standard ones to increase the chances of finding primers in the highly repetitive area (Agena Assay Design Suite v2.0, Agena Bioscience, USA). In total, only 13 out of 40 targeted SNPs passed quality control from the *in silico* assay design and were successfully genotyped in all 1,238 participants from the GABRIELA Ulm cohort (36). Genotyping was performed using the Sequenom MALDI-TOF mass spectrometry system in collaboration with the Institute of Clinical Molecular Biology, Kiel University.

#### Distinguishing Between *DEFB4A* and *DEFB4B* and Copy Number Variant Analysis

To determine the presence of one or the other gene copy and to distinguish between *DEFB4A* and *DEFB4B*, we selected 200 gender- and age-matched subjects from GABRIELA Ulm as an exploratory cohort equally distributed between different exposure and outcome groups (**Supplementary Table 2**). We developed RFLP (Restriction Fragment Length Polymorphism)-based assay to discriminate between *DEFB4A* and *DEFB4B*. First, we retrieved the sequences of *DEFB4A* and *DEFB4B* from the online genome browser Ensembl^4^ (37) (GRCh38.p12 primary assembly) and aligned them using the Basic Local Alignment Search Tool (BLAST, NCBI; U.S. National Library of Medicine, 8600 Rockville Pike, Bethesda MD, 20894 USA). We used commercially available restriction enzymes to digest only one of the copies after targeted PCR fragment amplification. We used the free online tool NEBcutter V2.0 (38) to identify suitable enzymes. We designed a primer pair framing a specific single nucleotide difference between the two gene copies: at this position, *DEFB4B* is cut by a specific restriction enzyme (Alu I, New England Biolabs, Germany) while *DEFB4A* remains uncut. As an internal positive control of the experiment, the primer pair also included two cutting sites in both gene copies. A standard PCR was carried out for each sample using a final DNA concentration of 1.3 ng/µL and annealing temperature of 61°C. The primer sequences were 5′-*TGTAATGAGCATTGCACCCAATAC*-3′ (forward) and 5′-*TCACAGTATAGGCTGGGCCTTA*-3′ (reverse). Digestion was conducted at 37°C for 10 h followed by enzyme-inactivation at 80°C for 1 h. After digestion, fragments from the two different gene copies were separated by agarose gel-electrophoresis (**Supplementary Figure 1A**). As a control for the accuracy of the RFLP method, we purified (ReliaPrep™ DNA Clean-Up and Concentration System, Promega, USA) and sequenced (Sanger sequencing, ThermoFisher Scientific, Germany) the 667bp (*DEFB4A*) and 445bp (*DEFB4B*) fragments from DNA of 43 healthy controls from an independent explorative study cohort called EXACT (39). Fragment purification from agarose gels was done by using the protocol for DNA purification from gel slices as provided by the manufacturer (Promega, USA). Sequencing revealed an accurate overlap of the fragments with the respective references, including the single nucleotide differences between the gene copies. The only discrepancy was detected in the area of the 9 bp insertion of *DEFB4A*, which appeared on the gel as a double band at the height of the 667 bp fragment. Therefore, the double band may indicate that the 9 bp insertion of *DEFB4A* is not present in both alleles (**Supplementary Figure 1A**).

#### Statistical Analysis

Deviation from Hardy-Weinberg equilibrium (HWE) was analyzed by chi-square test in the control group using PLINK version 1.0 (40). All markers were in HWE (p>0.0001). We generated Linkage Disequilibrium (LD) plots with Haploview (41) and performed all further analyses with R software (Version 3.0.1). Normally distributed data are presented as mean with standard deviation (SD) or 95% confidence interval (CI); non-parametric data as median and interquartile range (IQR). Differences between two groups were analysed using unpaired Wilcoxon or Student’s t-test depending on their Gaussian distribution. We evaluated associations of binary traits primarily by logistic regression, stratified for farmer status as previously described (33), using the *survey*-package in R statistics^5^. Odds ratios (OR), 95% CI and *p*-values are reported for association analyses. All *p*-values <0.05 were considered statistically significant as the analyses were hypothesis-driven. Only SNPs with a minimum of three minor allele carriers within the cohort were analyzed. To investigate whether the presence or absence of *DEFB4A* and *DEFB4B* was associated with health status, we applied chi-square tests.

### Murine Asthma Model Experiments

#### Mice

To assess preventive effects of hBD-2 in a model of inflammatory airway disease, we used female BALB/c mice between 7 and 8 weeks of age (Charles River, Italy), which received food and water *ad libitum* during the experiments. Animal-related research followed the 2010/63/EU and National legislation regulating the use of laboratory animals (Official Gazette 55/13) and the Institutional Committee on Animal Research Ethics (CARE-Zg). All animal experiments were performed according to the specifications of the senior investigators (MK, JW, PN) by Fidelta Ltd., Croatia.

#### Sensitization Procedure

For sensitization of the animals, a solution with 1 mg house dust mite (HDM) protein/ml saline was prepared according to the manufacturer’s instructions (33 mg HDM/vial; lot no 305469; Greer, USA). This solution was combined with equal amount (1:1 ratio) of complete Freund’s adjuvant (CFA) dissolved in PBS. The animals were distributed according to sensitization and treatment into three groups of 12 mice each (results section, **Figure 3**). Mice from group 1 received subcutaneous sensitization with saline containing CFA while groups 2 and 3 were sensitized subcutaneously with 100 µg HDM in 0.2 ml saline and CFA at day 0. Group 1 did not receive any treatment, while mice from group 2 received oral treatment with vehicle (0.5% carboxymethylcellulose) at days 12, 13, and 14, and mice from group 3 received oral hBD-2 at the same days. Sensitization on day 0 was applied with a 100 µl Hamilton glass syringe and a 16G cannula. According to the sensitization and treatment received, the groups were also called: Saline (1), vehicle/HDM (2) or hBD-2/HDM (3).

#### Preparation and Dosing of the Test Compound Human Beta-Defensin 2 (hBD-2)

Oral treatment with 1.2 mg/kg/day (0.4 mg/kg, 3 times a day) hBD-2 (Novozymes, Denmark) started at day 12, 2 days prior to the challenge with saline or HDM (day 14). The first daily dose was given at approximately 8 a.m. each day, followed by two additional administrations with 6-h intervals. The last dose was administered orally on day 14, 1 h prior to the challenge, at the volume of 10 ml/kg. The solutions for each dosing group had a concentration of 0.04 mg/ml. Concentration of the used hBD-2 ampules was confirmed with a CV% < 0.5 (triplicates) and purity was further verified to be 98.6% (UPLC). Endotoxins were below 0.004 EU/mg.

#### Asthma Induction and Prophylactic hBD-2 Administration

To induce allergic asthma, mice from groups 2 and 3 were immunized subcutaneously on day zero with 100 µg HDM in 0.2 mL saline per animal; group 1 received 100 μL of CFA dissolved in saline instead. On days 12 and 13, the mice received either vehicle (group 2) or hBD-2, 0.4 mg/kg, orally, three times a day (group 3). On day 14, only two doses of vehicle or hBD-2 were administered, respectively. One hour after the last dose, mice from groups 2 and 3 were intranasally challenged with 25 µg of HDM in 50 μL of saline. No additional treatments following challenge were carried out.

#### Airway Hyper-reactivity Measurements

At day 16, approximately 48 h after HDM application, 6 mice from each group were challenged with methacholine. Immediately after the challenge, lung resistance (cm H_2_O/mL/second) and dynamic lung compliance (mL/cm H_2_O) were automatically measured by a DSI’s Buxco^®^ FinePointe™ RC system (DSI, USA), and the results were computed by the RC system’s software. Each mouse was anesthetized (0.2% Xylazine plus 5 mg/mL Narkamon dissolved in saline) and tracheostomized after approximately 10 min for direct measurement of the respiratory flow and lung pressure. Mice were then loaded into a plethysmograph in a supine position, and a water or ethanol filled tube was placed two-thirds down the esophagus. For artificial ventilation, tracheal tubes were placed and fixed with tied suture, (stroke volume: body weight/100, rate: 120 breaths/min). Additionally, 0.1 mL of diluted ketamine (10 mg/mL) per 10 g body weight was added intraperitoneal to each mouse. For airway hyper-reactivity measurements, mice were initially exposed to aerosolized PBS for the baseline value, followed by increasing concentrations of 5 μL methacholine (3.125, 6.25, 12.5 and 25 mg/mL), nebulized for 3 min before lung function was recorded. The automated data acquisition software Finepointe™ (DSI’s Buxco) was used to continuously record various basic parameters, automatically generating resistance and dynamic compliance, and performing statistical analysis. The data were calculated at each time point, and lung resistance and compliance values were shown as a curve for each group.

#### Bronchoalveolar Lavage Fluid and Lung Cell Collection and Processing

Bronchoalveolar lavage fluid (BALF) was collected from all the animals (n=36) 48 h after HDM challenge. In brief, the trachea of each animal was cannulated, the lungs were washed with 3 volumes of cold PBS (0.4, 0.3 and 0.3 mL; 1 mL in total) and the collected BALF was placed into an Eppendorf test tube for each mouse. The tubes were centrifuged at 3,500 rpm and 4°C for 5 min, and cell pellets were re-suspended in 600 µL PBS by vortexing. Total and differential cell counts in BALF were determined on an automated haematological analyzer (Sysmex XT-2000iV).

After the bronchoalveolar lavage, the lungs of all animals were exposed and excised by opening the thorax, cutting down either side of the sternum and ribs, and trimming back. The lungs were removed from the thorax, snap frozen in liquid nitrogen and stored at −80°C until preparation of the homogenates for cytokine measurements. Frozen lungs were placed into PBS (1 volume tissue to 5 volumes PBS) containing a protease inhibitor cocktail (Roche, Switzerland) and homogenized using an IKA Turbo XT laboratory homogenizer (IKA, Germany). The tubes were subsequently centrifuged for 10 min at 12,000 rpm and 4°C. The clear supernatant was transferred to a new tube and stored at −80°C until further analysis. The concentration of seven asthma-related inflammatory cytokines, namely Interleukin (IL)-4, IL-5, IL-6, IL-9, IL-13, IL-33, and tumor necrosis factor (TNF)-α, was determined in lung homogenates of all mice using commercially available ELISA kits, following the manufacturer’s instructions (R&D systems, USA).

#### Statistical Analysis

For comparison of lung resistance and compliance, cell counts and cytokine levels from the different groups, we used one-way ANOVA with post-hoc t-test (Bonferroni corrected). Homogeneity of variances was asserted using Levene’s Test, which showed that equal variances could be assumed (all p-values > 0.24). Statistical analyses were performed using SPSS Statistics (IBM, USA). All *p*-values <0.05 were considered statistically significant.

## Results

### Mutations, Polymorphisms and Copy Number Variants in the hBD-2 Region Are Associated With Asthma and Atopy in Children

**Figure 1:**
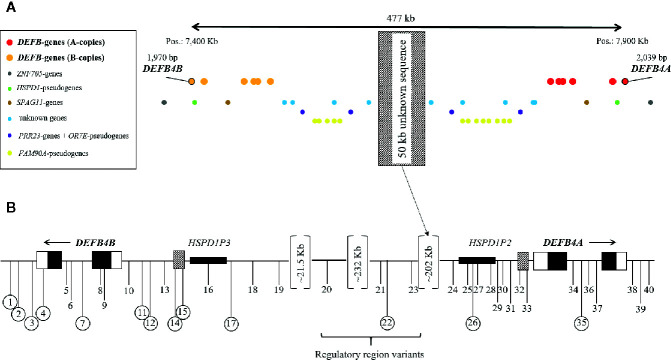
*DEFB4* locus on chromosome 8p23.1 with surrounding genes and pseudogenes **(A)** and location of the 40 selected SNPs for genotyping in GABRIELA Ulm **(B)**. **(A)** Red dots: A-copies of the β-defensin genes; orange dots: B-copies of the β-defensin genes; red and orange dots with black circle: *DEFB4* genes. Blue dots: unknown genes; yellow dots: *FAM90A*-pseudogenes; purple dots: *PRR23*-genes and *OR7E*-pseudogenes; brown dots: *SPAG11*-genes; green dots: *HSPD1*-pseudogenes; grey dots: *ZNF705*-genes. The hatched rectangle in the middle represents a not yet sequenced 50 Kb area, which presents like a mirror in this cluster. **(B)** positions of the selected SNPs for genotyping are shown as black vertical lines with numbers in relation to the position of *DEFB4A* and *DEFB4B*. Circled numbers represent SNPs that were successfully genotyped. Black boxes refer to exonic regions, white boxes to UTRs, hatched boxes to putative promoter regions.

The genes *DEFB4A* and *DEFB4B*, both coding for hBD-2, are located on chromosome 8 (chr8p23.1) in close vicinity to five other β-defensins (*DEFB103*, *DEFB104*, *DEFB105*, *DEFB106*, *DEFB107*), all of which are aligned in a peculiar, mirror-like cluster with their respective gene copies (**Figure 1A**). In the middle of this mirrored region resides a stretch of approximately 50 kb of DNA, which evaded all sequencing attempts so far due to massive repetition and duplication (42). *DEFB4B* locates approximately 477 kb upstream of *DEFB4A* in opposite reading directions, a general characteristic of β-defensins in this region. The structure and sequence of both *DEFB4A* and *DEFB4B* is highly similar (**Figure 1B**). The putative promoter region of *DEFB4A* reaches 1,612 bp upstream from the transcription start site and that of *DEFB4B* up to 1,874 bp. Overall, 61 out of 81 (75.3%) identified transcription factor binding sites of the region 2,000 bp upstream are identical. Exonic sequences are identical in 194 of 195 base pairs, leading to identical amino acid sequences, as the single nucleotide difference in exon 1 is a silent mutation. Intronic regions are 97.4% identical (1,629 out of 1,672 bp). The 3′ UTR and the first 48bp of the 5′ UTR from both genes match by 100%. However, the 5′ UTR of *DEFB4A* (84 bp) is 48bp longer than of the *DEFB4B* (36 bp).

Of the 458 SNPs annotated in the 500-kb region of interest in public databases (43), 40 were of interest for genotyping within 10 kb downstream of both genes, including the 477 kb between the genes, and followed the inclusion criteria described in detail in the methods section. From those, 17 were located in or very close to *DEFB4A*, 19 were described in or around *DEFB4B*, and four additional SNPs locate in different regulatory regions between the genes (**Figure 1**, **Supplementary Table 3**). Out of the 40 genetic variants [7 in high LD (LD>0.8)], we were able to genotype 13 and verify the existence of only 7 in our population with the applied settings. This low genotyping success rate was specific for this region, and thus contrasted with the high success rate in several other chromosomal regions genotyped at the same time, in the same cohort using identical design parameters (information available upon request). The complex nature of the region and the close-to-identical DNA sequence of the two genes could not be overcome by alternative genotyping methods applied. In case of a very low minor allele frequency (i.e., < 3 minor alleles) statistical analysis may lead to spurious results and thus, ORs and CIs were not calculated.

For the seven genetic variants we could confirm, minor allele frequencies and their association with asthma and allergy in our study population are shown in **Table 1**. Overall, MAF was magnitudes lower than expected when compared to CEU data. Four out of seven present genetic variants were extremely rare yet provided here for completeness. Indeed, all rare variants and two out of three more common polymorphisms showed associations with the outcomes under investigation (asthma and allergy, and the combination thereof), hence exceeding stochastic expectations. Due to the very low MAF, we could not assess the influence of farm exposure on these associations in stratified analyses as planned. *In silico* functional analyses showed that two associated variants (rs6651513 and rs543538872) have putative functional relevance: rs6651513 resides in an enhancer approximately 2kb upstream of *DEFB4B* and correlates with *DEFB4A* and *DEFB4B* expression, particularly in lung, skin, stomach, and esophagus mucosa (eQTL data provided by GTEx Portal^6^). Variant rs543538872 locates within a DNaseI Hypersensitivity Cluster 901 bp upstream of *DEFB4B* (44) (UCSC genome browser^7^), indicating that it could be part of the *DEFB4B* promoter region.

**Table 1:** Genetic associations of SNPs in or nearby *DEFB4A/B* with asthma and atopy in the GABRIELA Ulm cohort (n=1,238).

			MAF		Asthma		Atopy		Atopic asthma		Non-atopic asthma	
No.	SNP	Minor allele	CEU	GABRIELA Ulm	p-value	OR (95% CI)	p-value	OR (95% CI)	p-value	OR (95% CI)	p-value	OR (95% CI)
17	rs533344477	C	0.005	0	–	–	–	–	–	–	–	–
12	rs538653319	C	0.01	0	–	–	–	–	–	–	–	–
3	**rs538901702**	T	0.005	0.0004	**1.44^-26^**	NA	**5.67^-33^**	NA	**8.68^-30^**	NA	**4.59^-23^**	NA
11	**rs543538872**	T	0.015	0.002	**2.42^-90^**	NA	0.169	4.03 (0.55–29.28)	**9.14^-82^**	NA	**6.47^-91^**	NA
2	rs546946128	A	0.005	0	–	–	–	–	–	–	–	–
26	rs558364144	T	0.005	0	–	–	–	–	–	–	–	–
35	rs558912368	A	0.005	0	–	–	–	–	–	–	–	–
4	rs562192342	A	0.005	0	–	–	–	–	–	–	–	–
1	**rs562864847**	G	0.01	0.008	0.067	3.5 (0.91–13.41)	0.732	1.27 (0.33–4.87)	**0.0273**	4.79 (1.19–19.20)	**3.92^-181^**	NA
7	**rs567390989**	T	0.015	0.0008	0.077	12.19 (0.77–194.01)	0.344	4.2487 (0.21–84.70)	0.050	17.37 (0.99–304.04)	**4.38^-23^**	NA
22	**rs62640720**	G	0.328	0.0004	**1.3^-21^**	NA	**5.22^-28^**	NA	**1.57^-24^**	NA	**1.15^-18^**	NA
15	**rs6651513**	A	0.116	0.046	0.155	0.56 (0.25–1.24)	0.229	1.3935 (0.81–2.39)	0.4755	0.74 (0.32–1.69)	**0.001**	**0.08** **(0.02**–**0.35)**
14	rs73199779	C	0.056	0.028	0.839	0.92 (0.39–2.14)	0.281	0.6916 (0.35–1.35)	0.446	0.66 (0.23–1.92)	0.940	1.71 (0.50–5.92)

SNP, single nucleotide polymorphism; CI, confidence interval; MAF, minor allele frequency; NA, not applicable; OR, odds ratio.

Bold values represent significant association of the SNP with the respective disease.

**Figure 2:**
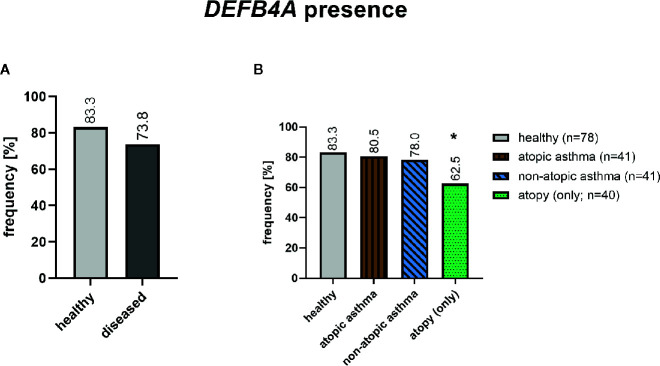
Presence of *DEFB4A* in healthy (n=78) and diseased (n=122) subjects after RFLP assay for *DEFB4A* and *DEFB4B*. The diseased group include 41 non-atopic asthmatics, 41 atopic asthmatics and 40 atopics. *p=0.012 (chi-square test).

Sequence alignment of the *DEFB4A* and *DEFB4B* showed a 98% sequence overlap with very few single nucleotide differences as mentioned above and depicted in alignment (**Supplementary Figure 1B**). To distinguish between the two almost identical genes, we used one single nucleotide difference in the intronic region (**Supplementary Figure 1C**). In total, 155 out of 200 subjects (77.5%) selected from our strata of interest from the GABRIELA Ulm cohort as described above (method section), have a *DEFB4A* gene. *DEFB4A* was found significantly less frequently in atopic individuals (p=0.012) and the same, but non-significant trend, was found in asthmatics (**Figure 2**).

### Prophylactic Oral Treatment With hBD-2 Reduces Effects of HDM Challenge in a Murine Asthma Model

**Figure 3:**
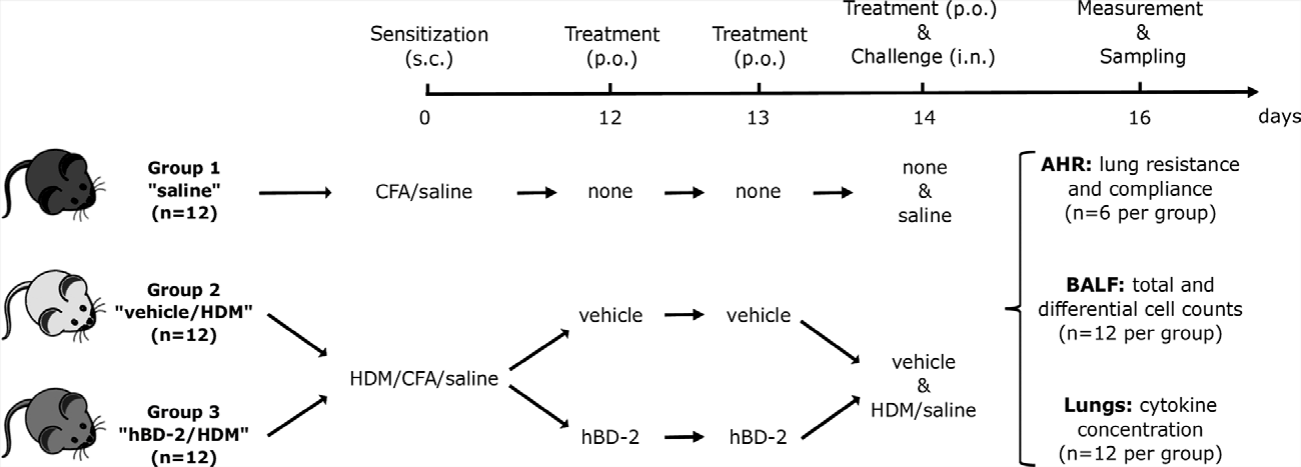
Prophylactic administration of hBD-2 in BALB/c mice and grouping according to sensitization and treatment. Challenge was carried out 1 h after the last dose of saline or vehicle on day 14. AHR, airway hyper-reactivity; BALF, bronchoalveolar lavage fluid; CFA, Complete Freund’s Adjuvant; hBD-2, human beta-defensin 2; HDM, house dust mite; *i.n.*, intra-nasal; *p.o.*, per oral; *s.c.*, subcutaneous.

As multiple associations between genetic variations in the *DEFB4A*/*DEFB4B* gene cluster and asthma and atopy in children suggest a role of these genes in asthma and allergy development, we investigated if hBD-2, due to its known functional entities, could help to explain protection against asthma and allergy by farm exposure and consumption of unprocessed milk on these farms. In an established HDM asthma mouse model, we studied the impact of orally administered hBD-2 (or vehicle only) prior to HDM challenge in 36 mice sensitized with either saline or HDM and assessed their lung hyper-reactivity and inflammatory response (**Figure 3**).

**Figure 4:**
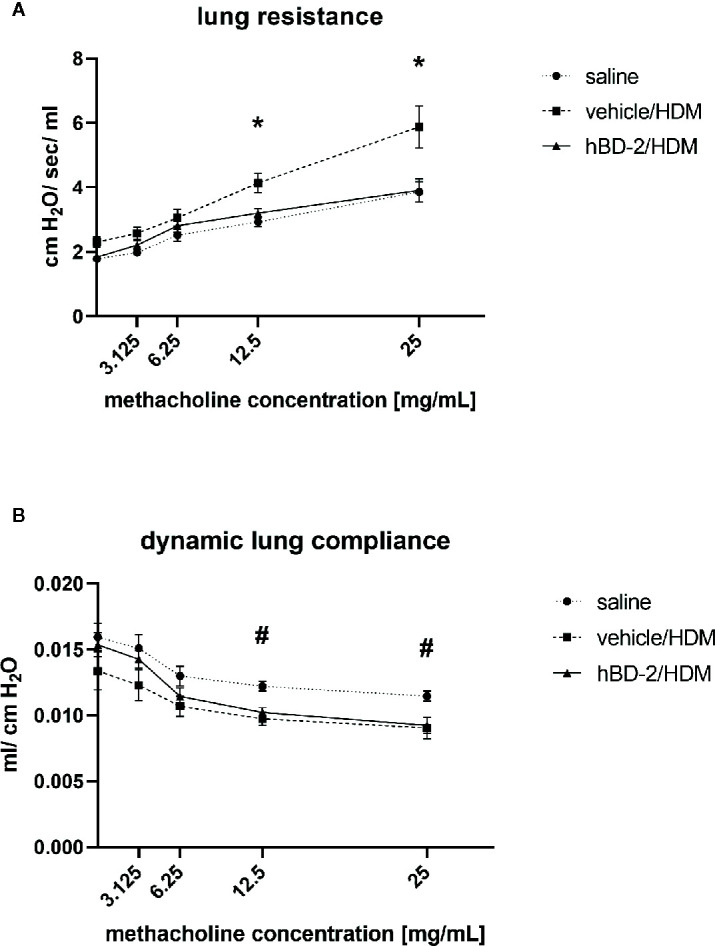
Lung resistance **(A)** and compliance **(B)** in 6 mice per group 48 h after saline or HDM challenge. Saline: Mice without treatment and challenged with saline only. Vehicle/HDM: Mice prophylactically treated with vehicle and challenged with HDM. hBD-2/HDM: Mice prophylactically treated with hBD-2 and challenged with HDM. *significant difference (ANOVA with post-hoc t-test; p<0.05) between vehicle treated and hBD-2–treated mice; ^#^significant difference (ANOVA with post-hoc t-test; p<0.05) between saline-challenged and HDM-challenged mice. hBD-2, human beta-defensin 2; HDM, house dust mite.

We assessed lung resistance and dynamic compliance between vehicle-treated (vehicle/HDM, group 2) and hBD-2-treated mice (hBD-2/HDM, group 3) after HDM challenge. Six animals per group initially received nebulization with PBS and subsequently with four different concentrations of methacholine (3.125, 6.25, 12.5 and 25 mg/mL). Vehicle-treated, HDM-challenged mice exhibited a dose-dependent increase in lung resistance paralleled by reduced lung compliance when compared with both unchallenged, and hBD-2 treated, HDM challenged mice (**Figure 4**). At the highest methacholine concentrations, the vehicle/HDM group showed significantly increased lung resistance compared to the hBD-2/HDM group (*p*=0.02 at 12.5 mg/ml and *p*=0.025 at 25 mg/ml). Notably, mice treated prophylactically with orally administered hBD-2 were fully protected against HDM-induced lung resistance, despite diminished lung compliance at the highest methacholine doses (**Figure 4**).

**Figure 5:**
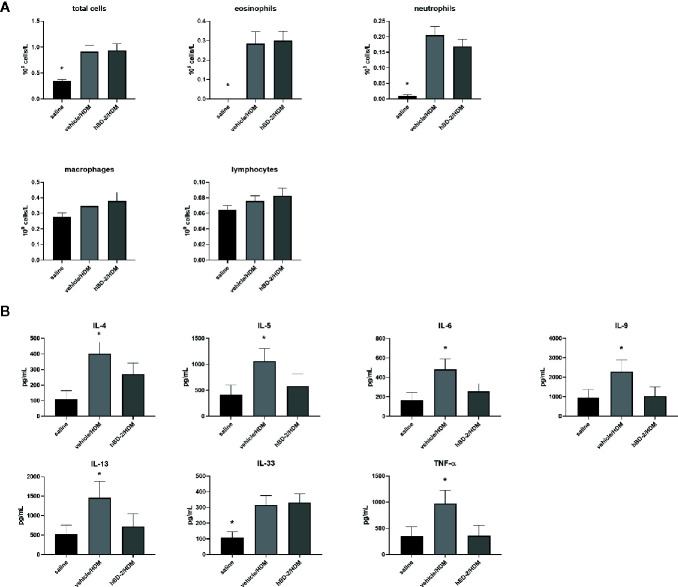
Total and differential cell counts (eosinophils, neutrophils, macrophages, and lymphocytes) in bronchoalveolar lavage fluid **(A)** and concentration of inflammatory cytokines (TNFα, IL-4, IL-5, IL-6, IL-9, IL-13, and IL-33) in lung homogenates of the 3 treatment groups (n=12 per group) **(B)**. Saline: mice sensitized with saline + CFA only (group 1). vehicle/HDM: mice sensitized with HDM at day 0, prophylactic treatment with vehicle at days 12 to 14, and challenge with HDM at day 14 (group 2). hBD-2/HDM: mice sensitized with HDM at day 0, prophylactic treatment with hBD-2 at days 12 to 14, and challenge with HDM at day 14 (group 3). *p < 0.05 when compared to other groups (ANOVA with post-hoc t-test,). hBD-2, human beta-defensin 2; HDM, house dust mite.

Total and differential cell count in BAL fluid was conducted 48 h after HDM challenge. The number of total and specific immune cells (eosinophils, neutrophils, macrophages and lymphocytes) was significantly increased after HDM challenge compared to saline-challenged mice, but no significant differences were observed between vehicle-treated and hBD-2-treated groups (**Figure 5A**). Thus, prophylactic oral treatment of HDM-sensitized mice with hBD-2 did not influence cell counts in BAL fluid after HDM challenge.

We next assessed hallmark asthma cytokines in lung homogenates to evaluate if oral hBD-2 treatment mitigated the pulmonary inflammation processes, hence explaining the relative protection against deteriorated lung function as described above. Vehicle-treated, HDM-challenged mice exhibited significantly enhanced tissue concentrations of all tested cytokines (IL-4, IL-5, IL-6, IL-9, IL-13, IL-33, and TNFα) compared to their PBS challenged counterparts (**Figure 5B**). Again, prophylactic oral administration of hBD-2 fully protected from HDM-induced cytokine release (with IL-33 being the sole exception) and thus significantly dampened pulmonary inflammation.

## Discussion

Genetic variations such as rare mutations, polymorphisms and copy number variations in the *DEFB4A*/*DEFB4B* genetic cluster associate with asthma and allergy in children. Furthermore, we provide evidence that hBD-2 could contribute to protective farm effects such as those mediated by the consumption of unprocessed cow’s milk (12) when prophylactic administration of oral hBD-2 mitigates pulmonary resistance and inflammation in a mouse model of HDM-induced asthma.

Investigating the locus harboring *DEFB4A* and *DEFB4B* specifically, we could confirm the high complexity of the region, which is truly at the frontier of the technical resolution currently possible in genetics. Numerous variants in the region evaded genotyping by different techniques and others showed dramatically lower MAF than predicted in the CEU population. These differences may only in part be explained by population characteristics but rather, are an expression of the high genetic complexity of the locus. As described in more detail below, the association with genetic variants in the hBD-2 locus for very comprehensible reasons did not allow to specifically perform an analysis stratified by farming exposure as originally planned.

The existence of two identical genes transcribing hBD-2 suggests a potential biological advantage in the capacity to mount a strong hBD-2 response if needed. This situation resembles the so called “cytokine gene cluster” on chromosome 5, containing the IL-4 and IL-13 coding genes, which are thought to have arisen from a gene duplication and also share a locus control region (45). Interestingly, IL-4 and IL-13 are both necessary and complementary for a type-2 immune response (46), as their functions have diverged over time, unlike that of the two (almost) identical genes coding for hBD-2. Preservation of genetic integrity in a coding sequence of a gene is always an active effort, which hints at the biological importance of the gene product. A similar situation exists for LL-37, an additional defensin (own unpublished data).

Subjects with atopy (with and without asthma) are more likely to lack *DEFB4A* in our study population. While all copy number analyses in previous studies did not discriminate between *DEFB4A* and *DEFB4B*, our approach explores for the first time the two copies separately. Despite copy segregation obtained by only a single nucleotide difference in the RFLP-like experiments, subsequent sequencing confirmed specificity for the respective copy. Still, it remains possible that *DEFB4A* is present in a mutated, hitherto unannotated form, and thus not identified in our analysis. In addition, some individuals in our study cohort may not simply miss *DEFB4A*, but have duplicated *DEFB4B*, similar to a homozygous state of a certain genotype (here: *BB* instead of *AB*). Further mutations and polymorphisms in the *DEFB4A*/*DEFB4B* cluster are also associated with a high likelihood to influence the regulation of gene expression according to *in silico* eQTL analyses. rs6651513 is located in an enhancer region 2 kb upstream of *DEFB4B* and it seems to negatively influence gene expression in the lung and skin and positively influence the expression in stomach, esophagus, and vagina, according to the online tool ensembl^8^. rs62640720 is also located in a regulatory region (CTCF binding site, in promoter) and positively influences the expression in vagina and skin, while the expression in stomach, esophagus, and lung are negatively affected. Assuming that increased expression of hBD-2 in the lungs protects against asthma and allergies, these two mutations seem to promote the development of asthma and allergies by decreasing hBD-2 expression.

Yet, our genetic data on mutations have to be interpreted with caution since some of the genotyped variants are extremely rare, not allowing to calculate odds ratios. In the context of all the evidence, including copy number variance and polymorphisms, the sum of all the effects is rather suggestive if not convincing. Taken together, these findings point toward the possibility that a lack, or inadequate production, of hBD-2 could lead to an increased susceptibility to asthma and allergy development, under the premise of a potentially protective effect of hBD-2 in these diseases.

Differences in copy numbers of the hBD-2 coding genes have been reported to correlate with other chronic inflammatory diseases. In the IBD, Morbus Crohn’s Disease, a decrease of hBD-2 gene copy numbers has also been found (47), whereas in psoriasis hBD-2 gene copy numbers seem to be increased (48). This corresponds well with the more recent observation that asthma and Crohn’s disease are comorbidities (49) and also share genetic traits (50) while such overlaps are not common with psoriasis. Interestingly, in comparison with psoriatic skin lesions, hBD-2 is reduced in the skin of patients with atopic dermatitis (51).

In addition to their shared lack of gene copies for the hBD-2 production, both asthma and Crohn’s Disease are influenced by the same environments: early exposure to farm environment is protective against the development of both (4–6, 10). We used the GABRIELA Ulm cohort, which is based on children living in rural areas, some with and some without farming contact, to explore the connection between mutations in the hBD-2 coding genes and asthma and allergy in a protective farm environment for the development of inflammatory diseases. Our previous studies have already suggested that genetic effects may be modified by environmental exposures and in combination have significant effects on the microbiome, explaining some of the protective farm effects (13, 33). However, in these studies the *DEFB4A* and *DEFB4B* genes were consistently neglected, as the genotyping chips used did not cover this genomic region. Unfortunately, the unexpectedly low MAF of all investigated genetic variants in the locus did not allow for further stratified analysis in our population due to its very low frequency, this kind of analysis is even beyond the resolution of much larger datasets. It would have been of interest to investigate gene by environment effects in individuals exposed and non-exposed to farming environments, but the absence of these analyses does not hamper the overall conclusion, that genetic alterations in a gene coding for a potential key player in the protective farming effect, is associated with asthma and allergy development in the general population. In contrast, this suggests that alterations of hBD-2 expression are associated with asthma development irrespective of protective environments.

As data from farm studies in asthma and allergy suggested an independent protective effect of unprocessed farm milk consumption, we hypothesized that if hBD-2 plays a role in this mechanism, hBD-2 production cannot only occur in the airways but also in the gut and based on the genetic data it should be increased to exert its protective function. As farm effects are only protective if they occur early in life, we further hypothesized that such increased hBD-2 production should occur prior to asthma development. As our initial goal was to investigate the potential protective effect of hBD-2 against asthma, we applied hBD-2 after sensitization and before challenge, which is also in line with standard protocols for asthma prevention experiments.

Our proof-of-concept mouse experiments support biological relevance of prophylactic hBD-2.

Specifically, prophylactic oral hBD-2 treatment of HDM-sensitized mice lowered the production of an array of classical asthma associated cytokines, namely TNF-α, IL-4, IL-5, IL-6, IL-9, and IL-13, in lung tissue of exposed mice subsequent to HDM challenge and concomitantly reduced lung resistance after methacholine-challenge. Since IL-4, IL-5, and IL-13 promote airway eosinophilia, mucus overproduction and bronchial hyper-responsiveness, it is likely that hBD-2 improves lung resistance indirectly by lowering those cytokines in the lung (52). Interestingly, hBD-2 had no effect on the cell counts in BALF, suggesting at least in this model, that its immune regulatory properties reflected *in situ* manipulation of pulmonary cytokine release rather than affecting immune cell influx. These data align well with a recent publication elucidating the immunomodulatory potential of hBD-2 in experimental colitis as well as in lipopolysaccharide-challenged mice and human peripheral blood mononuclear cells (17). Unaffected cell counts in BALF of hBD-2-treated mice seem exclusive to the oral administration route described here for the first time. However, one could speculate that the observed effect is due to possible impurities in the production process of the protein, but our analyses showed a high degree of purity. To completely rule out this possibility, an additional control group, treated with a peptide of similar but random amino acid composition like hBD-2, could be included in future experiments. While intranasal administration of hBD-2 even in a therapeutic setting lessens pulmonary inflammation in models of both steroid sensitive and steroid refractory asthma, treatment efficacy in those models was mirrored by diminished cell influx (27). Notably, intranasal administration of another antimicrobial peptide, mCRAMP (murine orthologue of LL-37), triggered asthma exacerbation in an allergic asthma mouse model (53), hence suggesting a unique role of hBD-2 in this setting.

Obviously, one cannot directly translate these findings from experimental mouse models to humans, and additional research is still necessary to enhance our understanding of how hBD-2 may affect asthma development in humans. Considering the respiratory challenges of asthmatic patients, it is nevertheless of significant relevance that oral administration of hBD-2 effectively mitigated pulmonary inflammation. Another future approach could be taken by applying hBD-2 even before sensitization to investigate a potential protective effect of hBD-2 against allergy development.

Future studies are needed to define the pertinent crosstalk between gut and lung in this setting and to establish if and how lung and gut microbiome signatures may affect disease trajectories. To this end, it was first shown in 2006 that colonic bacterial dysbiosis can alter the immune response of the lung after pulmonary infection in mice (54). It has further been demonstrated that constitutively expressed hBD-1 retains antimicrobial activity after proteolytic degradation by gastrointestinal proteases (55), while other defensins, e.g. HD5 and HNP4, not only preserve their antimicrobial activity upon degradation, but even enhance antimicrobial potency and specificity (56, 57). If this situation occurs in the context of orally administered hBD-2, and the extent to which such phenomenon would enhance transport of biologically active fragments from the gut to the lung, remains to be described.

It is also possible that the potent immunomodulatory nature of hBD-2 imprints systemic immunity to curb lung inflammation. Indeed, a recent report with detailed immune assessment of 514 infants 18 months of age corroborate exaggerated systemic immunity, in particular blood neutrophils and IL-13 producing T cells, at the time of enrollment to predict both transient and persistent childhood asthma (22). Early changes in the microbiome of children developing asthma and allergy have also been described recently (13). It is thus plausible that an aberrant and weak hBD-2 response either due to genetically determined limitations or a missing signal from the environment may play a role in both an abnormal development of immunity and the microbiome during childhood.

In conclusion, we demonstrate that hBD-2 and its coding genes, *DEFB4A* and *DEFB4B*, play an important yet underestimated role in the onset of asthma and atopy. Genetic alterations in the gene locus or absence of *DEFB4A* significantly associate with the prevalence of asthma and atopy in children while our mouse experiments clearly indicate that hBD-2 can have a prophylactic role in preventing features of allergic asthma. If and how these mechanisms could be used to intervene in the development of childhood asthma and allergy, still needs to be determined.

## Data Availability Statement

The original contributions presented in the study are included in the article/**Supplementary Material**, further inquiries can be directed to the corresponding author.

## Ethics Statement

The studies involving human participants were reviewed and approved by ethics committee of Ulm University Germany (104/06, 69/10, and 137/14). Written informed consent to participate in this study was provided by the participants’ legal guardian/next of kin.

## Author Contributions

Study design: NB, AT, PN, BJ, JW, JG, and MK. Data collection: NB, AT, PN, BJ, JG, AF, and MK. Analysis and data interpretation: NB, ES-V, VG, AT, BJ, and MK. Manuscript writing: NB, ES-V, AT, BJ, and MK. All authors contributed to the article and approved the submitted version.

## Funding

BJ was supported by the Lundbeck foundation (grant number: R232-2016-2425) and Novo Nordisk Foundation (grant number: NNF17OC0026698).

## Conflict of Interest

PN is employed by Defensin Therapeutics. PN and JW hold shares of Defensin Therapeutics. Defensin Therapeutics holds patents on treatment with defensins.

The remaining authors declare that the research was conducted in the absence of any commercial or financial relationships that could be construed as a potential conflict of interest.

## Footnotes

1
www.ensembl.org

2
https://www.ncbi.nlm.nih.gov/

3
https://genome.ucsc.edu/

4
www.ensembl.org

5
https://www.jstatsoft.org/article/view/v009i08

6
https://www.gtexportal.org/

7
https://www.genome.ucsc.edu/

8
www.ensembl.org
