# Supplementary material for: Human β-Defensin 2 Mutations Are Associated With Asthma and Atopy in Children and Its Application Prevents Atopic Asthma in a Mouse Model
Source: Front Immunol. 2021 Feb 25;12:636061. doi: 10.3389/fimmu.2021.636061 (PMC7946850; doi:10.3389/fimmu.2021.636061)
Supplement: Supplementary file 1 [file DataSheet_1.docx]

***Supplementary Material***

**Supplementary Tables**

**Supplementary Table 1. Demographic data of GABRIELA Ulm (n=1,238).**

| **n (%)** | **Farmers** | **Exposed non-farmers** | **Non-exposed non-farmers** |
| --- | --- | --- | --- |
| Age in years [mean (SD)] | 9.3 (1.18) | 9.5 (1.2) | 9.3 (1.15) |
| Female sex | 254 (53.6) | 201 (49.5) | 175 (48.9) |
| Asthma | 54 (11.4) | 72 (17.7) | 75 (20.9) |
| Atopy | 128 (27) | 152 (37.4) | 169 (47.2) |
| Atopic asthma | 29 (6.1) | 46 (11.3) | 58 (16.2) |
| Non-atopic asthma | 24 (5.1) | 26 (6.4) | 17 (4.7) |
| Healthy | 323 (68.1) | 227 (55.9) | 172 (48) |
| **total** | **475** | **405** | **358** |

SD, standard deviation.

**Note: As expected and reported previously, farmers differ significantly from non- exposed non-farmers in all health outcomes depicted here (p>0.001), except for non-atopic asthma (p=0.54).**

**Supplementary Table 2. Distribution of the participants from the GABRIELA Ulm cohort (N=1,238) according to presence of asthma/atopy and to farm exposure. Numbers and distribution of the Exploratory cohort (n=200) for the RFLP-like experiment are depicted for comparison in brackets**

|  | **Farmers** | **Exposed non-farmers** | **Non-exposed non-farmers** | **Total** |
| --- | --- | --- | --- | --- |
| **Non-atopic asthmatics** | 24 (17) | 26 (14) | 17 (10) | **67 (41)** |
| **Atopic asthmatics** | 30 (10) | 46 (16) | 58 (15) | **134 (41)** |
| **Atopics only** | 98 (13) | 106 (9) | 111 (18) | **315 (40)** |
| **Healthy** | 323 (33) | 227 (28) | 172 (17) | **722 (78)** |
| **Total** | **475 (73)** | **405 (67)** | **358 (60)** | **1,238 (200)** |

Farmers, children living on a farm; exposed non-farmers, children living in rural area and with contact to farm-environment; non-exposed non-farmers, children living in rural areas, but without contact to farm-environment.

**Supplementary Table 3:** **SNP selection based on location on chromosome 8p23.1 (DEFB4A / DEFB4B region) and minor allele frequency (MAF) in the CEU population (n=99) of the 1000 Genomes Project (Phase 3).**

| **#** | **SNP** | **Location on Chr8 (GRCh38p13)** | **Location (relative to gene)** | **Minor allele** | **MAF (CEU population)** | **Primer/Assay Design** | **plexed** | **Genotyping successful?** | **MAF (GABRIELA Ulm)** |
| --- | --- | --- | --- | --- | --- | --- | --- | --- | --- |
| **1** | rs562864847 | 7,413,561 | 1,294bp downstream (*DEFB4B*) | G | 0.01 | successful | yes | yes | 0.008 |
| **2** | rs546946128 | 7,414,592 | 263bp downstream (*DEFB4B*) | A | 0.005 | successful | yes | yes | 0 |
| **3** | rs538901702 | 7,414,654 | 201bp downstream (*DEFB4B*) | T | 0.005 | successful | yes | yes | 0.0004 |
| **4** | rs562192342 | 7,414,875 | 3’ UTR (*DEFB4B*) | A | 0.005 | successful | yes | yes | 0 |
| **5** | rs563436847 | 7,415,783 | Intron (*DEFB4B*) | A | 0.005 | rejected  (too many unknown bases 5' side of SNP) | no | no | NA |
| **6** | rs540005106 | 7,415,989 | Intron (*DEFB4B*) | T | 0.005 | rejected  (repetitive sequences surrounding) | no | no | NA |
| **7** | rs567390989 | 7,416,430 | Intron (*DEFB4B*) | T | 0.015 | successful | yes | yes | 0.0008 |
| **8** | rs536192108 | 7,416,780 | missense variant (Exon 1 of *DEFB4B*) | T | 0.03 | rejected  (repetitive sequences surrounding) | no | no | NA |
| **9** | rs71509106 | 7,416,786 | synonymous variant (Exon 1 of *DEFB4B*) | A | 0.455 | rejected  (repetitive sequences surrounding) | no | no | NA |
| **10** | rs563699591 | 7,417,301 | 438bp upstream (*DEFB4B*) | T | 0.005 | rejected  (repetitive sequences surrounding) | no | no | NA |
| **11** | rs543538872 | 7,417,764 | 901bp upstream (*DEFB4B*) | T | 0.015 | successful | yes | yes | 0.002 |

MAF=0, no minor allele present in the cohort; NA, not available; HWE, Hardy-Weinberg-Equilibrium.

**cont. Supplementary Table 3.**

| **#** | **SNP** | **Location on Chr8 (GRCh38p13)** | **Location (relative to gene)** | **Minor allele** | **MAF (CEU population)** | **Primer/Assay Design** | **plexed** | **Genotyping successful?** | **MAF (GABRIELA Ulm)** |
| --- | --- | --- | --- | --- | --- | --- | --- | --- | --- |
| **12** | rs538653319 | 7,417,778 | 915bp upstream (*DEFB4B*) | C | 0.01 | successful | yes | yes | 0 |
| **13** | rs181291950 | 7,418,603 | 1,740bp upstream (*DEFB4B*) | C | 0.005 | rejected  (repetitive sequences surrounding) | no | no | NA |
| **14** | rs73199779 | 7,418,737 | regulatory region (1,874bp upstream of *DEFB4B*) | C | 0.056 | successful | yes | yes | 0.028 |
| **15** | rs6651513 | 7,418,945 | regulatory region (2,082bp upstream of *DEFB4B*) | A | 0.116 | successful | yes | yes | 0.046 |
| **16** | rs4840753 | 7,420,012 | non-coding transcript exon variant of *HSPD1P3* (3,149bp upstream of *DEFB4B*) | G | 0.364 | rejected  (repetitive sequences surrounding) | no | no | NA |
| **17** | rs533344477 | 7,421,304 | 4,441bp upstream of *DEFB4B* (intergenic) | C | 0.005 | successful | yes | yes | 0 |
| **18** | rs371594216 | 7,423,381 | 6,518bp upstream of *DEFB4B* (intergenic) | A | 0.035 | rejected  (repetitive sequences surrounding) | no | no | NA |
| **19** | rs62636843 | 7,426,738 | 9,875bp upstream of *DEFB4B* (intergenic) | C | 0.313 | rejected  (repetitive sequences surrounding) | no | no | NA |
| **20** | rs9772875 | 7,448,293 | Intron (*SPAG11B*) | G | 0.035 | rejected  (repetitive sequences surrounding) | no | no | NA |

MAF=0, no minor allele present in the cohort; NA, not available; HWE, Hardy-Weinberg-Equilibrium.

**cont. Supplementary Table 3.**

| **#** | **SNP** | **Location on Chr8 (GRCh38p13)** | **Location (relative to gene)** | **Minor allele** | **MAF (CEU population)** | **Primer/Assay Design** | **plexed** | **Genotyping successful?** | **MAF (GABRIELA Ulm)** |
| --- | --- | --- | --- | --- | --- | --- | --- | --- | --- |
| **21** | rs529816867 | 7,680,629 | TF binding site (214,048bp upstream of *DEFB4A*) | T | 0.005 | successful | yes | no  (call rate=0) | NA |
| **22** | rs62640720 | 7,680,896 | regulatory region (213,781bp upstream of *DEFB4A*) | **G** | **0.328** | successful | yes | yes | **0.0004** |
| **23** | rs2954244 | 7,686,828 | regulatory region (207,849bp upstream of *DEFB4A*) | G | 0.283 | successful | yes | no  (call rate=0) | NA |
| **24** | rs201270314 | 7,888,776 | intergenic (5,901bp upstream of *DEFB4A*) | A | 0.136 | rejected  (repetitive sequences surrounding) | no | no | NA |
| **25** | rs200715941 | 7,891,377 | non-coding transcript exon variant of *HSPD1P2* (3,300bp upstream of *DEFB4A*) | C | 0.141 | rejected  (repetitive sequences surrounding) | no | no | NA |
| **26** | rs558364144 | 7,891,690 | non-coding transcript exon variant of *HSPD1P2* (2,987bp upstream of *DEFB4A*) | T | 0.005 | successful | yes | yes | 0 |
| **27** | rs71537819 | 7,891,722 | non-coding transcript exon variant of *HSPD1P2* (2,955bp upstream of *DEFB4A*) | G | 0.49 | successful | yes | yes, but not in HWE | 0.348 |
| **28** | rs71254900 | 7,892,553 | non-coding transcript exon variant of *HSPD1P2* (2,124bp upstream of *DEFB4A*) | C | 0.01 | successful | yes | yes, but not in HWE | 0.362 |

MAF=0, no minor allele present in the cohort; NA, not available; HWE, Hardy-Weinberg-Equilibrium.

**cont. Supplementary Table 3.**

| **#** | **SNP** | **Location on Chr8 (GRCh38p13)** | **Location (relative to gene)** | **Minor allele** | **MAF (CEU population)** | **Primer/Assay Design** | **plexed** | **Genotyping successful?** | **MAF (GABRIELA Ulm)** |
| --- | --- | --- | --- | --- | --- | --- | --- | --- | --- |
| **29** | rs144625140 | 7,892,590 | intergenic (2,087bp upstream of *DEFB4A*) | G | 0.177 | successful | yes | no  (call rate=0) | NA |
| **30** | rs76923789 | 7,892,711 | intergenic (1,966bp upstream of *DEFB4A*) | C | 0.152 | successful | yes | no  (call rate=0) | NA |
| **31** | rs571335647 | 7,892,792 | intergenic (1,885bp upstream of *DEFB4A*) | A | 0.005 | successful | yes | no  (call rate=0) | NA |
| **32** | rs9329210 | 7,892,867 | regulatory region of *DEFB4A* and *–B* in different tissues, e.g. lung and skin (1,810bp upstream of *DEFB4A*) | C | 0.273 | successful | yes | yes, but not in HWE | 0.186 |
| **33** | rs71244071 | 7,893,065 | regulatory region (open chromatin, 1,612bp upstream of *DEFB4A*) | T | 0.263 | rejected  (repetitive sequences surrounding) | no | no | NA |
| **34** | rs372478398 | 7,894,241 | intergenic (436bp upstream of *DEFB4A*) | A | 0.141 | rejected  (repetitive sequences surrounding) | no | no | NA |
| **35** | rs558912368 | 7,894,785 | Intron (*DEFB4A*) | A | 0.005 | successful | yes | yes | 0 |
| **36** | rs539952500 | 7,894,819 | Intron (*DEFB4A*) | A | 0.005 | rejected  (repetitive sequences surrounding) | no | no | NA |
| **37** | rs552320996 | 7,895,028 | Intron (*DEFB4A*) | C | 0.005 | rejected  (repetitive sequences surrounding) | no | no | NA |
| **38** | rs41507446 | 7,896,940 | 224bp downstream of *DEFB4A* | C | 0.02 | rejected  (repetitive sequences surrounding) | no | no | NA |
| **39** | rs72626630 | 7,898,039 | 1,323bp downstream of *DEFB4A* | C | 0.414 | rejected  (repetitive sequences surrounding) | no | no | NA |
| **40** | rs202117264 | 7,898,104 | 1,388bp downstream of *DEFB4A* | T | 0.293 | successful | yes | no  (call rate=0) | NA |

MAF=0, no minor allele present in the cohort; NA, not available; HWE, Hardy-Weinberg-Equilibrium.

**Supplementary Figures**


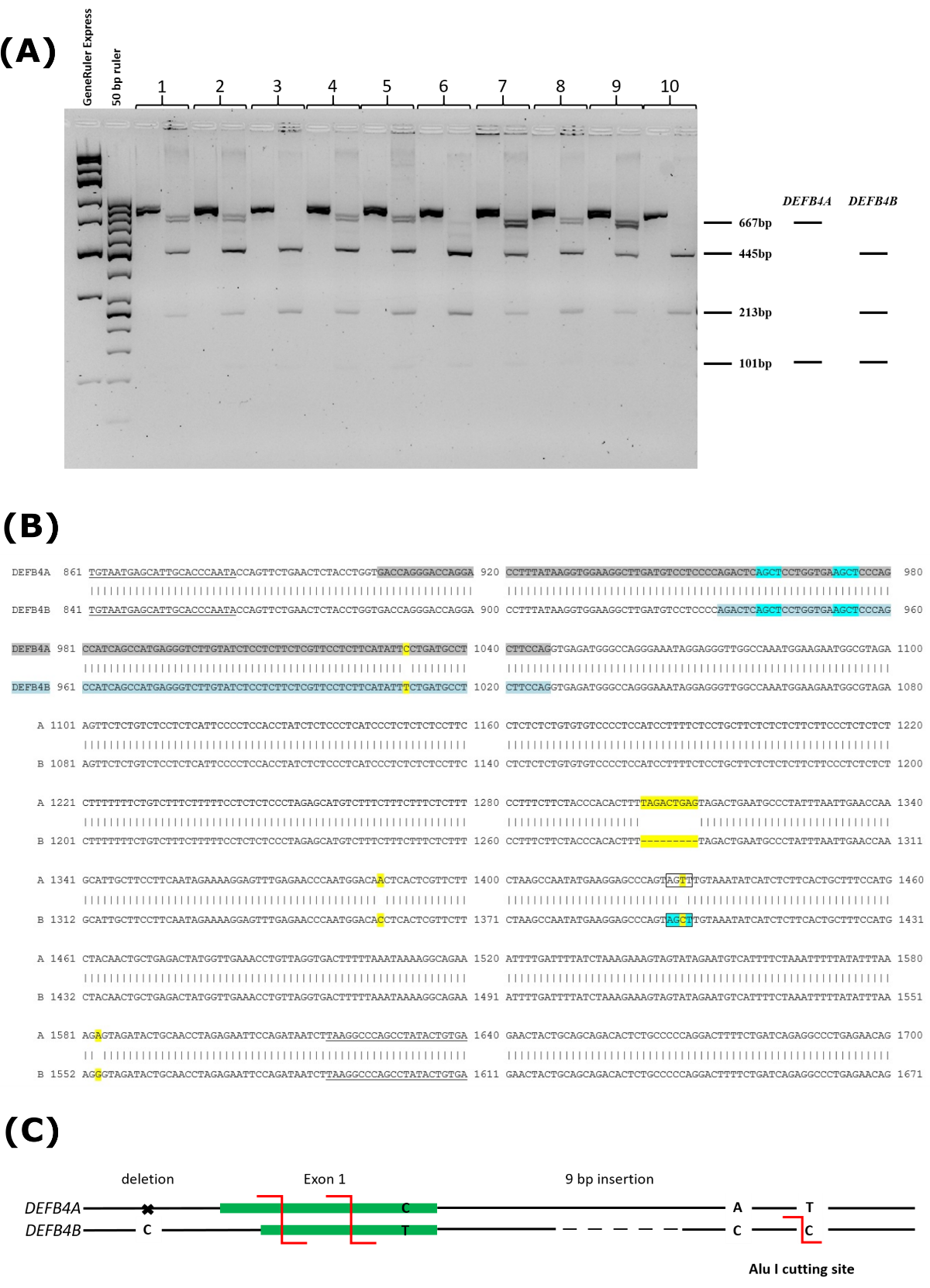


**Supplementary Figure 1. (A)** **Agarose gel after Alu I digestion.** 3.5% agarose gel after 2 hours gel-electrophoresis at 120V showing samples labelled 1 to 10. Left lane of each sample: undigested products (780bp for *DEFB4A* and 771bp for *DEFB4B*). Right lane of each sample: digested products; only the three biggest fragments (667bp, 445bp, and 213bp) are shown. **(B)** **DNA sequence alignment between *DEFB4A* and *DEFB4B*.** Only the first 840bp of *DEFB4A* and 831bp of *DEFB4B* are shown, respectively. Primers used for the RFLP-like assay are underlined. Differences in this section between A- and B-copy are marked yellow. Exon 1 is marked grey (*DEFB4A*) and dark blue (*DEFB4B*). Alu I cutting sites are bright blue. The cutting site distinguishing between the two gene copies is framed. **(C) Schematic alignment of *DEFB4A* and *DEFB4B* including the Alu I cutting sites (red marks).** Exons are shown as green bars. Solid lines represent identical sequences between the two genes. Dashed line represents missing sequence. Only nucleotides that are different are shown in the figure.


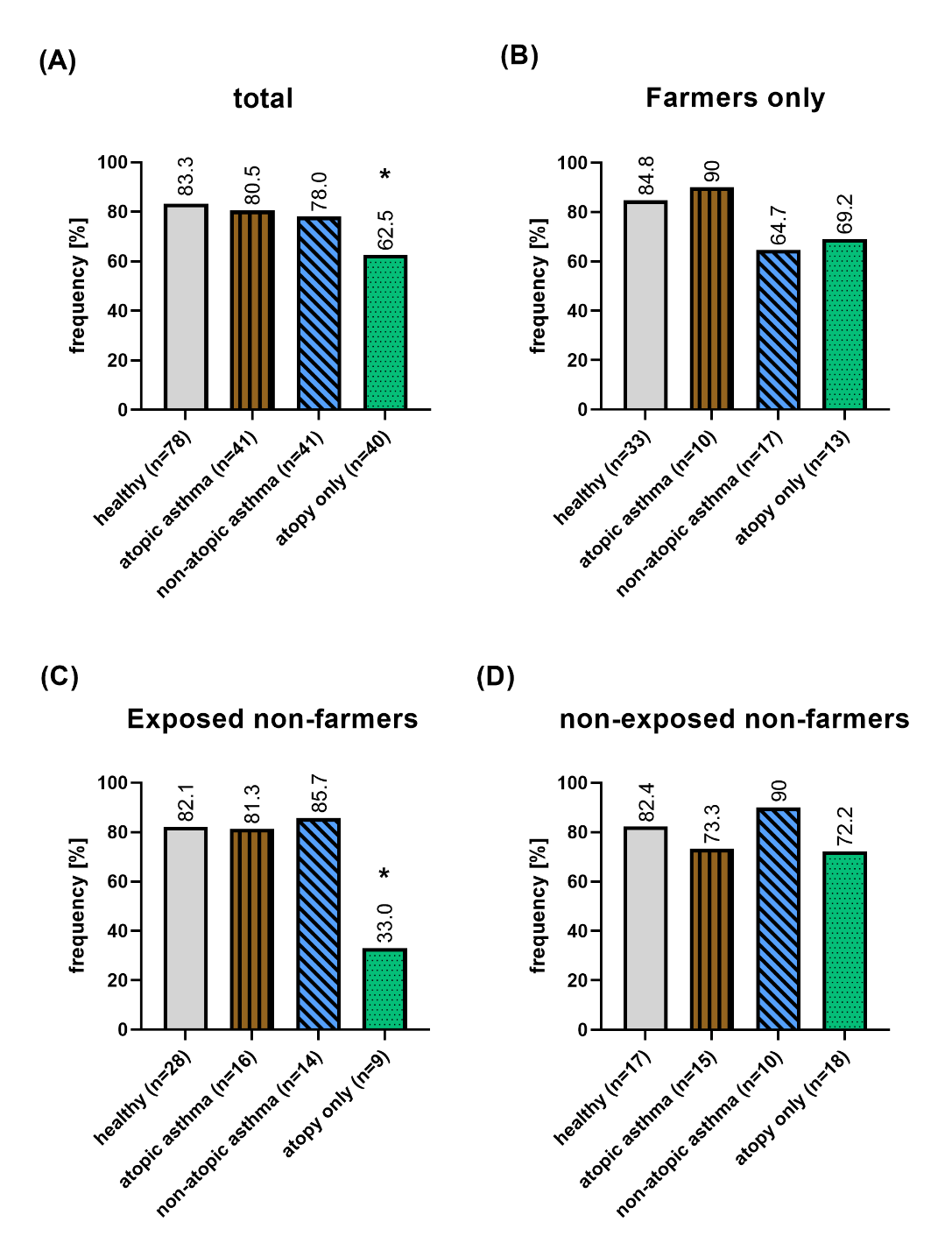


**Supplementary Figure 2:** **Presence of *DEFB4A* in GABRIELA Ulm subset (n=200) after stratification for farm status.** (A) *DEFB4A* in the whole subset. (B) *DEFB4A* in farmers only (n=73). (C) *DEFB4A* in exposed non-farmers (n=67). (D) *DEFB4A* in non-exposed non-farmers (n= 60).
